# Gut microbiota activity in chickens from two genetic lines and with outdoor-preferring, moderate-preferring, and indoor-preferring ranging profiles

**DOI:** 10.1016/j.psj.2022.102039

**Published:** 2022-07-03

**Authors:** Patryk Sztandarski, Joanna Marchewka, Paweł Konieczka, Żaneta Zdanowska-Sąsiadek, Krzysztof Damaziak, Anja B. Riber, Stefan Gunnarsson, Jarosław Olav Horbańczuk

**Affiliations:** ⁎ Institute of Genetics and Animal Biotechnology, Polish Academy of Sciences, Jastrzębiec, 05-552 Magdalenka, Poland; † Department of Animal Breeding, Faculty of Animal Breeding, Bioengineering and Conservation, Institute of Animal Science, Warsaw University of Life Sciences, 02-786 Warsaw, Poland; ‡ Department of Poultry Science and Apiculture, Faculty of Animal Bioengineering, University of Warmia and Mazury in Olsztyn, Oczapowskiego 5, 10-719, Olsztyn, Poland; § Department of Animal Science, Aarhus University, Aarhus DK-8830, Tjele, Denmark; # Department of Animal Environment and Health, Swedish University of Agricultural Sciences (SLU), S-532 23 Skara, Sweden

**Keywords:** free range, broiler, organic, microbiota activity

## Abstract

Despite the existing research into the gut microbiome of meat chickens, the associations between gut microbiome composition, its activity and chicken outdoor ranging frequency remain unexplored. The aim of this study was to determine the gut microbiota composition, activity and metabolic products in chickens of 2 different lines and 3 ranging profiles. Sixty non-beak trimmed birds, either Sasso or Green-legged Partridge were housed with access to outdoor ranges from wk. 5 to 10 of age. Outdoor ranges were video recorded to obtain frequencies of the birds’ range use. The information about relative abundance of selected bacterial groups in the ceca including *Lactobacillus* spp., *E. coli, Bifidobacterium* spp., and *Clostridium* spp. was obtained with the PCR method. Gut microbiota activity was assessed based on the glycolytic activity of bacterial enzymes including, α-glucosidase, β-glucosidase, α-galactosidase, β-galactosidase, and β-glucuronidase as well as based on the concentration of short-chain fatty acids (**SCFA**) in the caecal digesta. Statistical analysis was conducted by generalized linear mixed models, applying the breed and ranging profile as fixed effects and pen as a random factor. The lowest relative abundance of *Bifidobacterium* spp. was found in the cecal content of indoor-preferring Sasso birds (0.01 ± 0.001), as compared to all other birds in the experiment (ranging from 0.03 ± 0.01 to 0.11 ± 0.07; *P* = 0.0002). The lowest relative abundance of *E. coli* was identified for all outdoor-preferring birds and indoor- preferring Sasso birds (0.01 ± 0.001; *P* = 0.0087). Cecal activity of: α-glucosidase, β-glucuronidase and β-galactosidase was higher in Green-legged Partridges, as compared to Sasso (*P* = 0.013; *P* = 0.008; *P* = 0.004). Valeric acid concentrations were higher in moderate Green-legged Partridges than in Sasso of the same ranging profile (2.03 ± 0.16 vs. 1.5 ± 0.17; 0.016). The majority of the current results confirmed an effect of genotype and ranging profile on the various analyzed parameters. In outdoor-preferring birds, the consumption of pasture originating feed sources as a supplement to the indoor accessible cereal-based diet likely caused the positive effects on the birds’ microbial profile.

## INTRODUCTION

The chicken microbiome, defined as the entire environment of symbiotic, commensal, and pathogenic microorganisms present in the gastrointestinal tract (**GIT**), is important mainly for the digestion processes (Kogut, 2019). This is especially important in meat purpose chickens in commercial production, where higher efficiency is the main economic aim of the production.

There are more than 900 species of bacteria in the chicken gut microbiome (Binek et al., 2017). Not all off its characteristics and its functions have been well understood yet, but on the basis of the current knowledge, the composition of microorganisms inhabiting the chicken gastrointestinal tract is associated with gut morphology (Forder et al., 2007), health and immunity (Pan and Yu, 2014) or even behavior (van der Eijk et al., 2020) of the birds. For example, selected bacteria of the genus *Clostridium* (i.e., *Clostridium perfringens*) negatively affect the health of chickens. They might cause necrotic enteritis, and disrupt the proper functioning of the digestive system (Gharib-Naseri et al., 2019). On the other hand, bacteria of the genus *Lactobacillus* (i.e., *Lactobacillus acidophilus*) have a positive effect on bird health and performance supporting digestion and immune processes in the digestive system of chickens (Brisbin et al., 2011).

It has been found that the form, type and chemical composition of feed are closely linked to the gut microbiota activity. In the housing systems with outdoor access, the feed is not only available indoors, but the birds also find edible items on the ranges, like for instance insects, grass, herbs and stones, providing greater variety in feed forms and sources. Fermentable substances like: non-starch polysaccharides (**NSP**), starch and proteins that escape digestion and absorption in the upper part of the gut cause changes in the gut microbiota activity which is manifested in specific changes in SCFA concentrations, high β-glucuronidase activity and increased *E. coli*presence. Such high fermentation activity may be considered as detrimental to birds' health and performance (Konieczka et al., 2018). Production systems with outdoor access were found also to be associated with higher abundance of *Clostridium* spp*.* or *Lactobacillus* spp. in the chicken gut due to contact with soil and natural vegetation (Bjerrum et al., 2006; Hubert et al., 2019). Access to the range may alter the composition of the gut microbiota even due to weather factors like natural light or rain (Thaiss et al., 2016). The duration of time spent at the range may be as well important.

The relationships between the housing environment and the chicken microbiome composition and activity are also not fully explored, especially considering the various chicken production systems and genetic strains of birds, but research confirms that such relationships exist (Hubert et al., 2019; Kers et al., 2019; Ocejo et al., 2019). It is known that poultry housing environment influences microbiota diversity and structure (Kers et al., 2018). For instance, the presence of increased levels of ammonia in conventional poultry housing systems can permute infections caused by *E. coli* spread from the birds GIT to the environment (Landman et al., 2013).

The level of development of the GIT and its content have been found to be potential indicators of the chickens’ ranging profiles developed for classifying the frequency with which birds used the outdoor ranges (Marchewka et al., 2020) and forage consumption (Marchewka et al., 2021). Therefore, as the outdoor range use differs among individual birds, the composition and the gut microbiota activity may also potentially differ. Further investigations are necessary to confirm this.

Gut microflora activity and composition may be influenced by genetic factors. In a previous study we discovered significant differences between 2 genotypes of birds: slow-growing Sasso chicken and native Polish chicken Green-legged Partridge, mainly in digestive tract measurements for example, small intestine length, ceca length, colon length, and villus area. However, no previous studies have investigated microbiome activity in relation to ranging levels in slow-growing Sasso chickens and Green-legged Partridge chickens.

Understanding the associations of the ranging profiles of birds with different genetic background on their microbiota composition and activity is important to ensure the optimal health and welfare of the birds reared in housing systems with access to the outdoor ranges. This is particularly important nowadays as we can notice increased public concerns of animal welfare and higher attention of consumers toward meat from poultry reared in low-input systems.

The aim of this study was to determine the gut microbiota composition, activity and metabolic products in 2 genotypes of chickens, each with the three ranging profiles: outdoor-preferring, moderate-preferring and indoor-preferring (Marchewka et al., 2020). We hypothesized that the chickens which were identified as homogeneous in terms of ranging profile would show similar quantitative microbial composition of the same genus and similar gut microbiota activity, regardless of the breed.

## MATERIALS AND METHODS

The experiment took place in the Mazovian region of Poland, at the experimental farm of the Institute of Genetics and Animal Biotechnology of the Polish Academy of Sciences, in August and September of 2018. No Ethical Committee approval was required, as the current study was performed with no invasive experimental procedures applied to the animals during their life. In this experiment, mimicking the real on-farm production cycle, we did not perform any procedures exceeding standard husbandry procedures.

### Animals, Housing and Management

One hundred twenty non-beak trimmed mixed sex birds of each of 2 breeds (total n = 120 birds), Green-legged Partridge - indigenous Polish breed of heritage chicken and Sasso line C44 were used in the experiment. Sasso C44 is a commercially available, colored slow-growing hybrid of broilers (Hendrix Genetics BV, The Netherlands). Sasso birds are well skilled to forage on the outdoor ranges, having high resistance to low temperatures and diseases, while the meat is characterized by a very good taste and quality (Getiso et al., 2017). Sasso birds reach their slaughter weight of 2.3 to 2.8 kg at about 2 months of age. Until wk 5 of age, 120 birds were reared in the experimental facility without outdoor access in 2 pens, divided by the breed into 2 groups (1 group per pen) of 60 birds. At the age of 5 wk, all individuals were relocated from the rearing facility to the experimental house, both at the same location. Eight female and 2 male chickens were assigned to each single-breed group housed in 12 pens until 10 wk of age. In each pen, 6 birds (5 females and 1 male) with similar body weight within each breed (on average 2,030.6 ± 68.9 g for Sasso and 705.9 ± 8.5 g for Green-legged Partridge) were selected as focal animals. To make the recognition of individuals possible all birds were fitted with a laminated paper mark of the size of 9 cm high and 7 cm wide attached to the birds’ back by 2 elastic bands around its wings. Ten different colors of the marks were assigned in each pen randomly to the individual birds. Birds were wearing color mark during the entire experiment. They were inspected twice a day. No birds died during the experiment.

The outline of the experimental facilities has previously been presented in Marchewka et al. (2020) and Sztandarski et al. (2021). In short, the size of the indoor pens was 2.5 m × 3.5 m, resultingin a stocking density at slaughter age of 1.4 kg/m^2^ for Green-legged Partridge and 2.7 kg/m^2^for Sasso. Birds were housed on the sawdust litter, while in each pen, next to the wall there was a 0.5 m stripe covered with sand. Pens were cleaned when needed. In each pen, there were two 80-cm long wooden perches at 2 perching levels, one at the height of 15 cm and the second at 40 cm. The perching poles were 50 × 50 mm thick and had rounded edges. Each pen had direct access through the pophole (45 cm high × 50 cm wide) to an individual outdoor range (3.5 m × 30 m) providing 10.5 m^2^/chicken. All the outdoor ranges had the same vegetation coverage regarding botanical composition, no trees or shelters were present. The grass was mowed 1 wk. before the onset of the experiment. Each free-range area was provided with a semiautomatic bell drinker and a wooden box (1 m × 1 m) filled with sand.

The birds were habituated for 48 h to the new housing and social situation. Popholes were opened daily from 7.00 until 19.00 h. Commercial pelleted feed was used to nourish the birds. Feed and water were available ad libitum. The feed was composed of wheat, maize, sunflower expeller, pea, soybean expeller legumes mix, gruel corn, monocalcium phosphate, soybean oil, and calcium carbonate with supplements (Marchewka et al., 2020). The feed composition was intended to meet the birds’ nutritional requirements (Classen, 2017). No coccidiostats or other medication was used.

Birds were provided only natural light through uncovered windows. Light hours during the experimental period ranged from 12.7 h to 15.7 h/day. There was natural ventilation in the building. Indoor climate parameters were continuously collected by a device of the weather measuring device (Davis Instruments Vantage Pro 2 DAV-6152EU, CA) placed in the middle of the chicken rearing house at height of 1 m.

### Observations of Ranging Behavior

The behavioral data collection of range use in the current study has previously been described (Marchewka et al., 2020). Range use of the birds was recorded using video cameras. The 12 outdoor pens were video-recorded simultaneously and continuously using 6 cameras (BCS-DMIP2401IR-M-IV IP 4 Mpix), each covering 2 free-range areas. The cameras were attached to the wall of the experimental facility at a height of 3 m from the ground. The video material was recorded with the network recorder BCS-NVR0401-IP 4 channel BC. After that it was analyzed by one trained and experienced person, using the Chickitizer program (Sanchez and Estevez, 1998). From the recorded videos, 3 days were chosen per week of the experiment (5 wk.). On each of those days, 3 times of the day (at 8:00, at 13:00, at 18:00) a 3-min-period with 10 s sampling intervals was set and repeated after 10 min. The observer registered the absence or presence each of the experimental birds’ in the outdoor area.

### Sample Collection for Bacterial Composition and Activity Determination

At 72 d of life, birds from each group (n = 6) were sacrificed by cervical dislocation. Thereafter, the cavity was opened and both ceca were removed. The digesta from both ceca were collected and pooled in one test tube for each bird individually and was then divided into 3 portions to be used for different analysis. The collected digesta was immediately frozen in −80°C.

### Determination of Bacteria Relative Abundance

The relative abundance of selected bacterial groups in the caeca including *Lactobacillus* spp., *E. coli, Bifidobacterium* spp., and *Clostridium* spp. was performed using the PCR method. We modified Zhu et al. procedure to isolate bacterial genomic DNA from the cecal digesta (Zhu et al., 2002). Briefly; bacterial genomic DNA was extracted from digesta using the QIA amp. Fast DNA Stool Mini Kit (Qiagen, Stockach, Germany) according to the manufacturer's protocol. Then, the yield and purity of the isolated DNA were estimated spectrophotometrically (Nanodrop, NanoDrop Technologies, Wilmington, DE).

### Polymerase Chain Reaction Amplification of Bacterial 16S rRNA Gene

The primers and polymerase chain reaction (**PCR**) conditions used to amplify the bacterial 16S rRNA gene are shown in Table 1. The universal primer set was used to determine the total bacteria population. The detailed PCR conditions were set-up as previously reported for each respective bacteria group (Michalczuk et al., 2021). The obtained PCR-products were separated by electrophoresis on a 2% agarose gel. PCR products were quantified using ImageJ 1.47v software for densitometry measurements (National Institute of Mental Health, Bethesda, MD), with a density of bands for each bacteria group expressed in relation to the density of the total bacteria primers product. The density of the bands for each of bacteria group was expressed in relation to the density of the total bacteria primer product. Each sample was analyzed in duplicate.

**Table 1:** Sequences of primers used for amplification of bacterial 16S rRNA gene.

Bacterial group	Primers	Sequence 5’-3’	Base pair
Total bacteria	Forward	CGTGCCAGCCGCGGTAATACG	611
	Reverse	GGGTTGCGCTCTTGCGGGACTTAACCCAACAT	
*Lactobacillus spp.*	Forward	CATCCAGTGCAAACCTAAGAG	286
	Reverse	GATCCGCTTGCCTTCGCA	
*Escherichia coli*	Forward	GGGAGTAAAGTTAATACCTTTGCTC	585
	Reverse	TTCCCGAAGGCACATTCT	
*Clostridium spp.*	Forward	AAAGGAAGATTAATACCGCATAA	722
	Reverse	ATCTTGCGACCGTACTCCCC	
*Bifidobacterium spp.*	Forward	CGGGTGCTTCCCACTTTCATG	1417
	Reverse	GATTCTGGCTCAGGATGAACG	

### Bacterial Enzyme Activity

The activity of the gut microbiota was assessed based on the glycolytic activities of 5 bacterial enzymes in the cecal digesta including, α-glucosidase, β-glucosidase, α-galactosidase, β-galactosidase, and β-glucuronidase. Before the analysis, the digesta was thawed at 4°C for 3 h. The activity of the enzymes was determined spectrophotometrically according to Konieczka and Smulikowska, modified from Jurgoński et al. (Jurgoński et al., 2013; Konieczka and Smulikowska, 2018). To determine each specific enzyme we used: p-nitrophenyl-α-D-glucopyranoside for α-glucosidase, p-nitrophenyl-β-D-glucopyranoside for β-glucosidase, p-nitrophenyl-α-D-galactopyranoside for α-galactosidase, p-nitrophenyl-β-D-galactopyranoside for β-galactosidase, and p-nitrophenyl-β-D-glucuronide for β-glucuronidase (Sigma Chemical Co., St. Louis, MO).

### SCFA Concentration

The SCFA determination in the cecum digesta was performed according to the procedure described previously (Konieczka et al., 2018), using an HP 5890 Series II gas chromatograph (Hewlett Packard, Waldbronn, Germany) with a flame-ionization detector (**FID**) and a Supelco Nukol fused silica capillary column (30 m × 0.25 mm internal diameter, film 0.25 mm). Helium was employed as the carrier gas. The concentrations of individual SCFAs were estimated with an internal standard (isocaproic acid) using a mixture of standard solutions.

### Statistical Analysis

Birds of both breeds were divided into 3 ranging profiles using rank-frequency distribution (a discrete form of a quantile function in reverse order, giving the size of the element at a given rank) of their range use frequency summed over all the observation periods—that is, between 0 and 1,620 times. All the birds within a breed were assigned a rank based on their individual frequency of outdoor use. We segmented the rank distribution of the birds into 3 ranges: outdoor-preferring ranging profile, with the mean value of 506.1 ± 47.9 total outdoor uses per experiment per bird for Sasso and 502.6 ± 22.5 total outdoor uses per experiment per bird for Green-legged Partridge; moderate-outdoor ranging profile, with the mean value of 219.6 ± 18.8 total outdoor uses per experiment per bird for Sasso and 332.4 ± 13total outdoor uses per experiment per bird for Green-legged Partridge; and indoor-preferring ranging profile, with the mean value of 89.8 ± 11.7 total outdoor uses per experiment per bird for Sasso and 223.9 ± 12.1 total outdoor uses per experiment per bird for Green-legged Partridge. The rank intervals were equal (modified from Campbell et al., 2016).

Statistical analyses were performed with SAS 9.4. The GLIMMIX procedure was used to perform generalized linear mixed models for the microbiome composition, activity and metabolic products using either normal or gamma distribution where appropriate, applying the ranging profile group, breed and their interaction as fixed effects in the model. The pen was included in the model as a random effect. The assumptions of homogeneity of variance and normally distributed residuals were examined visually using the conditional Studentized residuals plots. The results are shown as means with standard errors, and *P*-values below 0.05 were considered significant, while between 0.05 and 0.06 were considered a significant trend. Tukey's post hoc test was performed to investigate significant differences between test groups.

## RESULTS

### Bacteria Composition

Effects of breed, ranging profile and their interaction on the relative abundance of selected bacteria in the ceca are presented in Table 2.

**Table 2:** Effects of breed, ranging profile, and their interaction on the relative abundance of selective bacteria in the caeca.

		DNA abundance			
Factors		*Clostridium* spp.	*Lactobacillus* spp.	Bifidobacterium spp.	*E. coli*
Breed					
	Sasso (n = 36)	0.32±0.03^A^	0.17±0.03	0.06±0.01	0.01±0.01^B^
	Green-legged Partridge (n = 36)	0.24±0.03^B^	0.14±0.02	0.07±0.02	0.04±0.01^A^
Ranging profile					
	Indoor-preferring	0.24±0.03	0.14±0.03	0.06±0.03	0.02±0.01^BA^
	Moderate-preferring	0.30±0.04	0.13±0.02	0.05±0.02	0.04±0.02^A^
	Outdoor-preferring	0.30±0.03	0.19±0.03	0.08±0.02	0.01±0.00^B^
Breed*ranging profile					
	Sasso*indoor-preferring	0.23±0.05	0.15±0.05	0.01±0.00^B^	0.01±0.00^C^
	Sasso*moderate-preferring	0.39±0.05	0.14±0.04	0.08±0.03^A^	0.03±0.02BAC
	Sasso*outdoor-preferring	0.33±0.05	0.21±0.04	0.07±0.03^A^	0.01±0.00^C^
	Green-legged Partridge*indoor-preferring	0.25±0.05	0.12±0.03	0.11±0.07^A^	0.04±0.02^BA^
	Green-legged Partridge*moderate-preferring	0.22±0.05	0.13±0.02	0.03±0.01^BA^	0.05±0.03^A^
	Green-legged Partridge*outdoor-preferring	0.26±0.05	0.18±0.04	0.09±0.03^A^	0.01±0.00^C^
		*P-*value			
Breed		0.0493	0.5018	0.1952	0.0074
Ranging profile		0.3109	0.3333	0.1715	0.0016
Breed*ranging profile		0.1501	0.9822	0.0002	0.0087

A-C Different letters within factor indicate significant differences (If the *P* value is < 0.05).

An effect of the interaction between breed and ranging profile was identified for the relative abundance of *E. coli* (*P* = 0.0087) and *Bifidobacterium* spp. (*P* = 0.0002). The lowest relative abundance of *E. coli* was identified for outdoor-preferring Sasso and Green-legged Partridges and indoor-preferring Sasso birds. The lowest relative abundance of *Bifidobacterium* spp. was found in the intestinal content of indoor-preferring Sasso birds as compared to all other birds in the experiment. The effect of breed was observed in the *Clostridium* spp. relative abundance (*P* = 0.0493): it was higher in Sasso chickens, as compared to Green-legged Partridges.

No significant differences were identified between ranging profiles of either Sasso or Green-legged Partridges regarding bacterial relative abundance.

### Microbial Enzymes Activity

Effects of breed, ranging profile, and their interaction on the microbial enzymes activity are presented in Table 3.

**Table 3:** Effects of breed, ranging profile, and their interaction on the microbial enzymes activity.

		Cecal digesta enzymes activity				
Factors		α - GLUCOSIDASE 400 nm	β - GLUCOSIDASE 400 nm	α - GALACTOSIDASE 400 nm	β - GLUCURONIDASE 400 nm	β - GALACTOSIDASE 420 nm
Breed						
	Sasso (n = 36)	1.26±0.06^B^	0.78±0.04	1.82±0.03	0.87±0.05^B^	5.53±0.29^B^
	Green-legged Partridge (n = 36)	1.47±0.05^A^	0.85±0.04	1.82±0.01	1.16±0.06^A^	6.77±0.19^A^
Ranging profile						
	Indoor-prefering	1.34±0.07	0.83±0.05	1.86±0.05	0.94±0.07	6.00±0.35
	Moderate-prefering	1.34±0.09	0.77±0.05	1.80±0.03	1.01±0.08	6.04±0.36
	Outdoor-prefering	1.41±0.06	0.85±0.04	1.81±0.01	1.08±0.07	6.36±0.28
Breed*ranging profile						
	Sasso*indoor-prefering	1.31±0.08	0.84±0.06	1.87±0.06	0.93±0.08	5.88±0.41
	Sasso*moderate-prefering	1.09±0.12	0.67±0.04	1.76±0.06	0.82±0.11	5.08±0.58
	Sasso*outdoor-prefering	1.35±0.12	0.79±0.08	1.79±0.02	0.81±0.03	5.30±0.59
	Green-legged Partridge*indoor-prefering	1.46±0.13	0.76±0.09	1.84±0.01	0.98±0.10	6.50±0.62
	Green-legged Partridge*moderate-prefering	1.52±0.11	0.84±0.07	1.82±0.01	1.14±0.11	6.71±0.36
	Green-legged Partridge*outdoor-prefering	1.44±0.06	0.88±0.04	1.82±0.01	1.22±0.09	6.88±0.22
		*P-*value				
Breed		0.013	0.229	0.535	0.008	0.004
Ranging profile		0.504	0.363	0.382	0.937	0.805
Breed*ranging profile						
		0.175	0.143	0.603	0.338	0.579

A-C Different letters within factor indicate significant differences (If the *P* value is < 0.05).

No effect of the interaction between breed and ranging profile was observed for any of the investigated enzymes activities. However, there was an effect of the breed on 3 of the enzymes that is, α-glucosidase (*P* = 0.013), β-glucuronidase (*P* = 0.008), and β-galactosidase (*P* = 0.04), where higher activity was observed in Green-legged Partridges, as compared to Sasso chickens.

No significant differences were identified between ranging profiles of either Sasso or Green-legged Partridges regarding microbial enzymes activity.

### SCFA

Effects of breed, ranging profile and their interaction on the SCFA concentration are presented in Table 4.

**Table 4:** Effects of breed, ranging profile, and their interaction on the short chain fatty acids (SCFA) concentration.

		SCFA (μmol/g)						
Factors		Acetic acid	Propionic acid	Isobutyric acid	Butter acid	Isovaleric acid	Valeric acid	Total SCFA
Breed								
	Sasso (n = 36)	62.13±2.52	20.63±1.04	2.02±0.13	9.90±0.87	1.84±0.11^A^	1.72±0.08	98.24±3.77
	Green-legged Partridge (n = 36)	67.80±2.49	21.67±0.97	2.00±0.12	9.34±0.44	1.63±0.12^B^	1.86±0.08	104.29±3.78
Ranging profile								
	Indoor	60.77±2.89	19.05±1.14	2.02±0.19	9.01±0.76	1.70±0.12	1.70±0.10	94.25±4.28
	Moderate	64.00±3.11	22.23±1.35	2.00±0.16	9.75±1.11	1.85±0.19	1.82±0.13	101.65±4.94
	Outdoor	69.42±3.11	21.96±1.12	2.01±0.10	10.04±0.66	1.67±0.12	1.83±0.08	106.93±4.45
Breed*ranging profile								
	Sasso*indoor	60.58±2.94	18.97±1.30	2.11±0.23	8.83±0.84	1.83±0.13	1.73±0.11^BA^	94.04±4.37
	Sasso*moderate	56.08±1.69	21.72±2.20	1.78±0.14	10.47±2.53	1.76±0.24	1.50±0.17^B^	93.31±5.71
	Sasso*outdoor	72.26±7.75	22.92±2.32	2.08±0.19	11.54±1.69	1.96±0.30	1.94±0.16^BA^	112.7±10.55
	Green-legged Partridge*indoor-prefering	61.58±9.76	19.38±2.63	1.64±0.17	9.79±2.01	1.16±0.13	1.57±0.13^BA^	95.11±14.38
	Green-legged Partridge*moderate-prefering	69.49±4.61	22.59±1.78	2.15±0.24	9.26±0.81	1.91±0.28	2.03±0.16^A^	107.42±7.10
	Green-legged Partridge*outdoor-prefering	68.00±2.80	21.49±1.25	1.97±0.13	9.29±0.46	1.53±0.10	1.78±0.09^BA^	104.05±4.23
		*P*-value						
Breed		0.303	0.985	0.615	0.449	0.030	0.526	0.659
Ranging profile		0.079	0.196	0.729	0.718	0.159	0.356	0.140
Breed*ranging profile		0.076	0.778	0.123	0.484	0.072	0.016	0.194

A-C Different letters within factor indicate significant differences (If the *P* value is < 0.05).

An effect of the interaction between breed and ranging profile was identified only for valerian SCFA (*P* = 0.016). The observed concentration of valerian SCFA was higher for moderate-outdoor Green-legged Partridges, as compared to moderate-outdoor Sasso chickens. An effect of breed on the isovalerian concentration was observed (*P* = 0.03), being higher in Sasso as compared to Green-legged Partridge chickens.

No significant differences were identified between ranging profiles regarding SCFA concentrations.

## DISCUSSION

Birds reared with access to the pasture consume material found outdoors, such as plants, insects, and stones. In our previous study we found that the frequency of range use by the chicken was associated not only with the ingested material, but also with the development of the bird gut and those associations differed between Green-legged Partridges and Sasso birds (Marchewka et al., 2021). However, it has not until now been investigated whether the relationship between outdoor range use and chicken gut microbiota exists.

The aim of this study was to investigate microbiota: selected main bacterial species presence, microbial enzymes activity, and SCFA concentration in the ceca (the main site of fermentation) of chickens with 2 different genotypes and 3 free-ranging profiles: outdoor-preferring, moderate-outdoor and indoor-preferring (Marchewka et al., 2020). The birds were divided into ranging profiles within each breed based on the frequency of the range use. Both breeds were well adapted to the rearing systems with outdoor access (Marchewka et al., 2020). Nevertheless, differences in the range use exist on the individual level, even if equal opportunity of outdoor access is provided (Rodriguez-Aurrekoetxea and Estevez, 2016).

The chicken intestinal microbiome contains several taxa. Non-pathogenic *Campylobacter* spp. or *E. coli* may be present in concentrations up to 10^7^ colony-forming units per gram (cfu/g) in the chicken intestine (Stern et al., 1995). Bacteria present in the GIT of chickens at lower concentrations are *E. coli*. Broiler chickens, especially in conventional housing systems are frequently infected with *E. coli*, which often results in disease and high economic losses, yet healthy poultry birds possess an innate resistance to infections (Moharrery and Mahzonieh, 2005). Certain strains of *E. coli* may, however, causes opportunistic secondary infections in poultry birds (Gross, 1990).

The analysis of the bacteria species relative abundance in the ceca of the birds in the current study showed the presence of the interaction between the genotype and ranging profile in 2 cases: *E. coli* and *Bifidobacterium* spp. relative abundance. The lowest relative abundance of *E. coli* was identified for outdoor-preferring Sasso and Green-legged Partridges and indoor Sasso birds. The lower abundance of *E. coli* identified in indoor-preferring Sasso chickens in the present study could suggest that the main reservoir of *E. coli* was found outdoors at the free ranges. In the case of the low *E. coli* abundance in outdoor-preferring birds, regardless of the genetic background, it can be suspected that it was associated with their frequent presence outside. Cereals commonly used in chicken diet are not only the source of valuable nutrients, but also contain antinutritional factors such as non-starch polysaccharides (**NSPs**), which reduce digestion and the level of peptides that exert beneficial effects on gut physiology, including the microbiome (Shakouri et al., 2009; Torok et al., 2011; Kers et al., 2018; Chen et al., 2019). In outdoor-preferring birds the consumption of pasture originating feed sources as a supplement to the indoor accessible cereal-based diet may have had a positive effect on the birds’ microbial profile.

Moreover, the lowest relative abundance of *Bifidobacterium* spp. was found in the caecal content of indoor-preferring Sasso birds, as compared to all other birds in the experiment. *Bifidobacteria* produce lactic and acetic acids in large amounts and take part in the stabilization of the gastrointestinal barrier, modulation of the local and systemic immune responses, inhibition of the pathogenic invasion and promotion of the bioconversion of unavailable dietary compounds into bioactive healthy molecules (Rossi and Amaretti, 2010). Some strains of *Bifidobacterium* spp. have been found to prevent *E. coli* colonization in the mouse GIT, where the main mechanism of this action was via acetic acid synthesis by *Bifidobacterium* spp. strains, resulting in the reduction of the luminal pH (Asahara et al., 2004). This potential inhibitory role of *Bifidobacterium* spp. in indoor-preferring Sasso birds is, however, contradictory to low abundance of *E. coli* found in the same birds. Nevertheless, studies are needed to explain the mechanisms ruling the abundance of bacteria strains in indoor-preferring Sasso birds, which could help to improve those birds' health and optimize their welfare, while potentially promoting range use.

The genotype of the chickens in this study affected *Clostridium spp.* relative abundance, being higher in Sasso chickens as compared to the Green-legged Partridge. In some circumstances it may indicate unfavorable microbiome features in Sasso chickens, as some poultry pathogens belong to the larger *Clostridium* spp. group. For instance *Clostridium perfringens* may cause necrotic enteritis (Olkowski et al., 2008). On the other hand, dietary supplementation *Clostridium butyricum* had positive effects on the growth, immune response, gut microbiota, and intestinal barrier function of broilers (Li et al., 2021).

The significant effect of the interaction between a genotype and ranging profile was found only on the concentration of one SCFA, where the highest concentration of valeric acid was observed in moderate-outgoing Green-legged Partridges. Microbial communities perform an important role in the growth and gut health by producing SCFA (Dunkley et al., 2007), modulating the morphological structure of the intestinal tract (Shakouri et al., 2009), and consequently influencing nutrient digestion and absorption (Choct, 2009). The indigestible carbohydrates, in which pasture diet is rich, in the gut can be used and converted into SCFAs by the microbial communities in broilers (Józefiak et al., 2004). Their concentrations are used as biomarkers of microbiota development and microbial-host interactions (Liao et al., 2020). The concentration and types of fermentation products formed by gut bacteria depend on the relative amounts of each substrate available, bacteria species and fermentation strategy of bacteria involved in the fermentation process (Liao et al., 2020). For example, chicken diet components like cereal type influenced the fermentation process and had an impact on SCFA presence and concentration (Józefiak et al., 2004). Valeric acid glyceride esters, added to the feed, promoted broiler performance, positively affected the morphology of the small intestinal mucosa and reduced the incidence of necrotic enteritis (Onrust et al., 2018). Previously, in moderate-outgoing Green-legged Partridges the weight of the pasture matter in the crop was 3 times higher, as compared to moderate-outgoing Sasso, and there was significantly more pasture matter identified, as compared to other ranging profiled birds of that breed (Marchewka et al., 2021). Hence, it can be suspected that the higher concentrations of valeric acid in moderate-outgoing Green-legged Partridges were associated with the pasture matter-rich diet those birds had, supporting the favorable microbiota composition. However, the direct associations between the diet, intestinal tract health, and gut microbial composition in birds of various genetic backgrounds allowed access to the outdoor pastures are yet to be discovered.

The activity of some investigated bacterial enzymes has been shown to differ between genotypes, primarily based on the type of ingested feed as demonstrated in poultry nutritional studies (Hübener et al., 2002; Shakouri et al., 2009; Zdunczyk et al., 2014; Konieczka and Smulikowska, 2018; Chen et al., 2019; Konieczka et al., 2020). In the present study the activity levels of the 3 investigated bacterial enzymes, including α-glucosidase, β-glucuronidase and β-galactosidase were decreased in Sasso birds, as compared to Green-legged Partridges. Within the commensal intestinal microbiota, species with the potential to improve poultry performance are particularly important, as they are also involved in cross-relation between the microbiota, gut epithelium and immune system, providing resistance to enteric pathogens (Konieczka et al., 2019). Those probiotic species contribute to an increase in the activity of many bacterial glycolytic enzymes, such as α-galactosidase, which hydrolyses dietary α-galactosides (RFO and other oligosaccharides); β-galactosidase, which contributes to the hydrolysis of β-galactosides; and α- and β-glucosidase, which contribute to the hydrolysis of NSPs (cellulose, β-glucans; Hübener et al., 2002; Zdunczyk et al., 2014). The enhanced activity of some bacterial enzymes, particularly β-glucosidase and β-glucuronidase, may be detrimental to the bird's health (Jin et al., 2000; Konieczka et al., 2018). It is worth to pay attention to the current results, since the increased activity of β-glucuronidase may also be indicative of increased proliferation of pathogenic bacteria in the gut, and it is associated with the higher risk of toxic and carcinogenic substances generation from nontoxic glycosides (Beaud et al., 2005).

Higher isovaleric acid levels were observed in Sasso as compared to Green-legged Partridges, regardless of the ranging profile. The genetic background of the host has been recognized previously as a factor that might influence gut microbiota composition (Schokker et al., 2015; Han et al., 2016). Increased production of isovaleric acid, which belong to the putrefactive SCFA are indicative of unfavorable conditions in the gut, including increased shifts in pathogenic bacteria and increased ammonia production (Koh et al., 2016). When comparing 2 breeds used in the current study, Sasso growth rates are much higher due to intensive genetic selection on this trait, as compared to Green-legged Partridges. The average slaughter body weight of roosters is around 2.5 kg and hens around 1.7 kg, which is achieved at about 5 mo of age (Krawczyk, 2009; Siwek et al., 2013). In comparison, Sasso birds reach a slaughter weight of 2.3 to 2.8 kg at about 2 mo of age (Getiso et al., 2017). In broilers (Arbor Acres male broilers), the concentration of isovalerate has previously been identified as increasing with the age of the birds (Liao et al., 2020). Therefore, the identified effects of the genetic background on the isovaleric acid concentrations could reflect the higher growth rates characteristic to Sasso birds. Higher concentrations of isovaleric acid in Sasso chickens may also indicate a poorer intestinal health resulting in poorer birds’ welfare, which require further attention. Finally, the study design, where birds were reared in breed-specific groups, could influence the results to some extent, as other studies reported that birds housed together show less variation of the gut microbiota, known as the cage effect (Meyer et al., 2012; Zhao et al., 2013; Chen et al., 2019), which may have wiped out the ranging profile effect.

Gut microbiome profile and diversity are closely linked to ensuring the health of the poultry used for meat production. Microbiome functions include protection against pathogens, nutrients production, and host immune system maturation (Stanley et al., 2014). Better health of the birds and optimal adaptation of their genotype to the housing systems with outdoor access safeguards their high welfare but also high productivity (Aruwa et al., 2021). Therefore, good understanding of the host-microbiome relationship remains integral. In the current study, some important knowledge gaps have been identified. In outdoor-preferring birds’ consumption of pasture originating feed sources as a supplement to the indoor accessible cereal-based diet may have positive effects on the birds’ microbial profile. However, there is not much known yet about the potential protective role of providing outdoor access to the birds in order to reduce *E. coli* levels in the gut and avoid secondary infections. Finally, the full interactions between the diet and intestinal health in birds of various genetic backgrounds with access to the outdoor pastures are yet to be discovered.

## CONCLUSIONS

Our hypothesis that the chickens which have been identified as homogeneous in terms of ranging profile will show similar quantitative microbial composition of the same genus and similar gut microbiota activity regardless of the breed was partially confirmed. The lowest relative abundance of *E. coli* was identified for outdoor-preferring Sasso and outdoor-preferring Green-legged Partridges. Therefore, in outdoor-preferring birds, consumption of pasture originating feed sources as a supplement to the indoor accessible cereal-based diet may have positive effects on the bird's microbial profile. Furthermore, we found significant effects of the genotype on the various parameters analyzed. Nevertheless, direct links between the diet, and gut microbial composition and intestinal health in birds of various genetic backgrounds that had access to the outdoor pastures are yet to be discovered.
